# On the Mechanical Properties of Microfibre-Based 3D Chitinous Scaffolds from Selected Verongiida Sponges

**DOI:** 10.3390/md21090463

**Published:** 2023-08-24

**Authors:** Tomas Duminis, Marcin Heljak, Wojciech Święszkowski, Alexander Ereskovsky, Izabela Dziedzic, Marek Nowicki, Martyna Pajewska-Szmyt, Alona Voronkina, Stefan R. Bornstein, Hermann Ehrlich

**Affiliations:** 1 Centre for Advanced Technologies, Adam Mickiewicz University, Uniwersytetu Poznańskiego 10, 61-614 Poznan, Poland; izadzi@amu.edu.pl (I.D.); marek.nowicki@amu.edu.pl (M.N.); mpszmyt@amu.edu.pl (M.P.-S.); 2 Faculty of Materials Science and Engineering, Warsaw University of Technology, ul. Wołoska 141, 02-507 Warsaw, Poland; marcin.heljak@pw.edu.pl (M.H.); wojciech.swieszkowski@pw.edu.pl (W.Ś.); 3 Institut Méditerranéen de Biodiversité et d’Écologie Marine et Continentale (IMBE), Aix Marseille Université, Station Marine d’Endoume, Rue de la Batterie des Lions, 13007 Marseille, France; alexander.ereskovsky@imbe.fr; 4 Faculty of Chemistry, Adam Mickiewicz University, Uniwersytetu Poznańskiego 8, 61-614 Poznan, Poland; 5 Department of Pharmacy, National Pirogov Memorial Medical University, Vinnytsya, Pirogov Str. 56, 21018 Vinnytsia, Ukraine; voronkina@vnmu.edu.ua; 6 Institute of Electronics and Sensor Materials, TU Bergakademie Freiberg, Gustav Zeuner Str. 3, 09599 Freiberg, Germany; 7 Department of Medicine III, Universitz Hospital Carl Gustav Carus, Technische Universitat Dresden, Fetschelstrasse 74, 01307 Dresden, Germany; stefan.bornstein@uniklinikum-dresden.de; 8 Departmen of Experimental Diabetology, German Institute of Human Nutrition Potsdam-Rehbruecke, 14558 Nuthetal, Germany

**Keywords:** tissue scaffolds, *Aplysina aerophoba*, Verongiida, demosponges, mechanical properties, 3D scaffolds, chitin, bromotyrosine

## Abstract

Skeletal constructs of diverse marine sponges remain to be a sustainable source of biocompatible porous biopolymer-based 3D scaffolds for tissue engineering and technology, especially structures isolated from cultivated demosponges, which belong to the Verongiida order, due to the renewability of their chitinous, fibre-containing architecture focused attention. These chitinous scaffolds have already shown excellent and promising results in biomimetics and tissue engineering with respect to their broad diversity of cells. However, the mechanical features of these constructs have been poorly studied before. For the first time, the elastic moduli characterising the chitinous samples have been determined. Moreover, nanoindentation of the selected bromotyrosine-containing as well as pigment-free chitinous scaffolds isolated from selected verongiids was used in the study for comparative purposes. It was shown that the removal of bromotyrosines from chitin scaffolds results in a reduced elastic modulus; however, their hardness was relatively unaffected.

## 1. Introduction

Quite frequently, the sheer complexity of producing a biocompatible 3D matrix; the high cost, which makes it unattractive for the industry; and the high risk of short- or long-term toxicity and chemical incompatibility between the different components in synthetic materials opens new areas of research for discovering alternative, nature-derived “ready-made” biomaterials.

**Figure 1:**
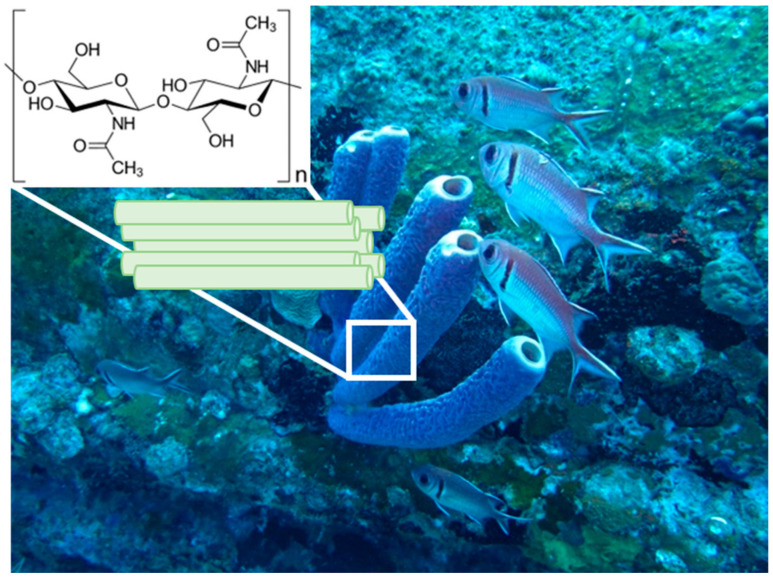
An underwater image of 30 cm-long marine demosponges belonging to the Verongiida order in their original environment. (Photograph courtesy: Dr. V. Ivanenko).

Structural polysaccharide chitin has been recently recognised as such a kind of 3D structured biological material that is excellently applicable within the “scaffolding strategy” of modern biomaterialogy [1]. In particular, chitinous constructs isolated from diverse representatives of cultivated marine demosponges, which belong to the Verongiida order (Figure 1), remain to be candidates with high potential in biomedicine [2,3] and bioinspired materials science [4].

Chitin found in verongiids forms highly intricate tubular three-dimensional (3D) skeletal structures with fibre diameters of up to 120 µm [5] with a great potential in biomaterials science, which has already found applications as tissue scaffolds [6,7,8] and as local drug delivery devices [9,10]. Non-toxicity, cell adhesion, and proliferation for various cell types (i.e., murine fibroblasts Balb/3T3, human dermal fibroblasts NHDF, human keratinocytes HaCaT, and human neuronal cells SH-SY5Y) were recently reported by Machałowski et al. [11] for chitinous skeletons derived via alkali-acid treatment of an *Aplysina fistularis* marine demosponge.

Recently, systematic “express” methods [12] have been developed to isolate poriferan 3D scaffolds of chitin in less than an hour [13,14,15]. This has opened further investigations into the mechanical properties of these 3D scaffolds for potential exploitation in tissue engineering and technology. To our best knowledge, there is still only one publication [11] where compressive theoretical modulus of 0.5 kPa for poriferan chitin has been reported.

However, the mechanical properties of biomaterials, including chitin, are quite important, particularly if they are used to regenerate body areas which require load-bearing or stiffness, such as hard tissues including bone and tooth [16,17].

As with any other material, the physiochemical properties of chitin can usually be linked to the various structural parameters on a molecular level and the various arrangement modes, such as the size, distribution, and shape it takes up, e.g., hollow tubular capillaries or a homogenous matrix. Chitin is found in nature as α-chitin (anti-parallel chains), β-chitin (parallel chains), and γ-allomorph (anti-parallel and parallel chains) in respect to the position of the reducing terminus sugar molecule [18]. Chitin mostly exhibits a nano- and microfibril arrangement through H-bonding and a sheet structure.

The bonding in this special molecule is the key to understanding the physical properties of this natural polymer. Individual N-acetyl-D-glucosamine units are covalently bound to one another via the β-(1-4)-glycosidic linkages to form a polymer. Intermolecular H-bonding C-O…NH between the polymer chains significantly influences the mechanical properties of chitin [19].

The different polymorphs of chitin may exhibit slightly altered mechanical performances as a result of structural differences in the availability of sites for H-bonding. A highly significant study by Sawada et al. [20] reported corresponding neutron diffraction experiments and showed direct experimental evidence of hydrogen-bonding positions of anhydrous β-chitin and found that three major hydrogen bonds are intramolecular O3–H···O5 and intermolecular O6–H···O7 and N2–H···O7 [20].

It is noteworthy to mention that there are some critical differences between α-chitin and β-chitin [21]. It is generally accepted that both the α and β polymorphs of chitin exhibit a strong chain network dominated by intra-chain hydrogen bonds between the groups of C═O⋯NH and C═O⋯OH. In the α-chitin conformation, additional inter-chain hydrogen bonds bind the hydroxymethyl groups, which is absent in the β conformation due to differences in the chain alignment. The H-bonding in γ-allomorph is relatively similar to that of α-chitin. The extensive H-bonding is also confirmed by recent experimental studies [20] and theoretical simulations [22].

It has been recently postulated [23] using electron density functional theory and molecular dynamics simulations that the acetyl group found in chitin, as opposed to its deacetylated counterpart, chitosan, may play a very significant role in determining superior mechanical properties observed within chitin. The authors Cui et al. [23] postulated that this causes more high-occupancy H-bonds along the inter-sheet direction of the chitin model. Additionally, the van der Waals interaction within chitin crystals is significantly enlarged due to the larger molecular mass of the acetyl group, the authors explain, which is also responsible for the differences in the mechanical properties observed between chitin and chitosan. This report is consistent with the results of an experimental study examining β-chitin with a similar microfibril arrangement where the degree of deacetylation was also found to influence the mechanical properties, such as the maximum stress and Young’s modulus, which decreased when reducing the deacetylation, and maximum elongation increased when decreasing the deacetylation [24].

A computational study by Wei and co-authors [21] revealed that the α-chitin crystal exhibits superior mechanical performance in response to tensile and shear loading. When a small-strain uniaxial tension is applied along the chain direction, the α-chitin crystal shows an elastic modulus at 48 GPa, almost twice as high as that of the β-chitin crystal at 27 GPa. Moreover, the shear modulus and strength of the α-chitin crystal are superior to those of the β-chitin crystal.

Besides chitin, diverse marine organisms, including sponges, also contain biominerals and pigments within their skeletal structure [19]. In the case of Verongiida order-related sponges, numerous bromotyrosines have been reported [25]. The biological role of these compounds was also suggested [26] to be protection against chitinase activity by microorganisms, which rely on the digestion of the chitinous matrix and use it as a source of carbon. Both the antiviral [27] and antibacterial activity of bromotyrosines are well-recognised [2]. The corresponding mechanisms of the aforementioned activities have been recently represented and discussed [10].

However, the role of this unique derivative of amino acid in the crosslinking of chitin in marine organisms, such as the Verongiida sponges, is poorly understood. Therefore, it can be suggested that the content of bromotyrosine, as well as the chemical interplay between chitin and bromotyrosine compounds, may play a very significant role in influencing the mechanical properties of chitin found in the verongiid sponges.

The mechanical properties of chitin films can be correlated to the amount of shrinkage from the gel to the final film [28]. Therefore, to retain flexibility, reduce dimensional distortion, and provide superior mechanical integrity in the dry state, it is important to manage the coagulation and shrinkage process during the preparation of chitin materials. For the production of flexible chitin films with thicknesses of 25–80 μm, cold-press processes (e.g., dissolution of chitin in dimethylacetamide-5% LiCl solution at 0 °C) have been used. To remove the solvent residue, the samples have been heated at 50 °C for 12 h and rinsed in 95% ethanol. The Young’s modulus of these flexible chitin films varied from 1240 to 3650 MPa, with a tensile strength between 38 and 60 MPa and transparency of up to 90% [28]. Chitin films with such mechanical properties have been suggested as very useful for wound dressing applications [28]. For a comparison of several mechanical properties between the relevant biomaterials, the reader is presented with Table 1.

Duan et al. [34] recently developed strong chitin-based transparent films with gas-barrier properties. The creation of these chitin-based films was based on dissolving chitin in aqueous 11 wt% NaOH and 4 wt% urea at a low temperature. At a thickness of 800 nm, they demonstrated transmittance of 87% and possessed excellent tensile strength of up to 111 MPa.

The high mechanical strength and Young’s modulus (higher than 150 GPa) of chitin-based materials is thought to be caused by the antiparallel extended crystal structure of chitin nanofibres. Due to such mechanical properties, chitin nanofibres can improve the mechanical properties of polylactide [35], poly (ε-caprolactone) [36,37], and acrylic resins [38,39].

Gadgey and Bahekar [31] recently used chitin from Philippine blue swimming crabs (*Portunus pelagicus*) and produced films using 5% (*w*/*v*) lithium chloride/N,N–dimethylacetamide (LiCl/DMAc) solvent. It was found that chitin polymer films have a tensile strength up to 44.22 MPa higher than the commercial plastic control samples which showed a tensile strength of 18.90 MPa. Moon et al. [40] recently analysed collagen/chitin composites produced using two polymorphs, α-chitin and β-chitin, and found that the β-chitin polymorph (parallel) exhibited much better mechanical properties due to the rearrangement of crystalline regions and formation of intermolecular hydrogen bonds with collagen.

Nonetheless, although the films developed from industrial, mostly crustacean chitin usually show good mechanical properties, they do not exhibit specifically interconnected microtubular 3D fibrous architectures. Consequently, the present investigations aim to understand the mechanical properties of alternative nature-derived 3D chitinous scaffolds isolated from the selected marine sponges, which possess α-chitin [19]. Based on molecular dynamics simulations, it has been reported that water can significantly influence the elasticity of simulated chitin-protein composites [41]. Therefore, the experiments herein were designed in a systematic way, having two types of samples: wet samples for the assessment of the elastic modulus (compressive) in the wet condition and dried embedded samples for the nanoindentation experiments. Both experiments assessed two types of samples: decellularized bromotyrosine-containing chitin scaffolds and decellularized and bromotyrosine-free chitin scaffolds. This was done to simultaneously determine the possible influence of bromotyrosines on the mechanical properties of poriferan chitin scaffolds.

## 2. Results

### 2.1. Monotonic Compression Test

**Figure 2:**
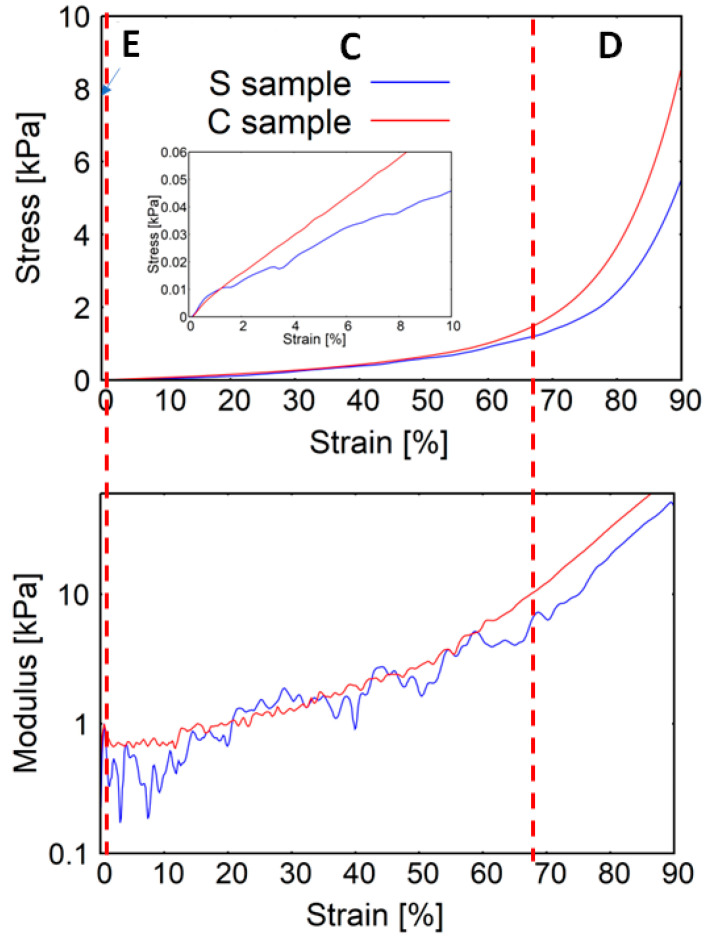
Stress–strain and modulus–strain curves of the investigated *A. aerophoba* sponge scaffolds. Three distinctive stages of compression were indicated: E—elastic stage, C—collapse stage, and D—densification stage. S samples (blue) correspond to bromotyrosine-containing samples and C (red) corresponds to bromotyrosine-free samples.

Figure 2 reports the stress–strain and the tangent modulus–strain curves for samples of chitinous scaffolds isolated from an *Aplysina aerophoba* demosponge cultivated under marine ranching conditions, tested under wet conditions.

**Figure 3:**
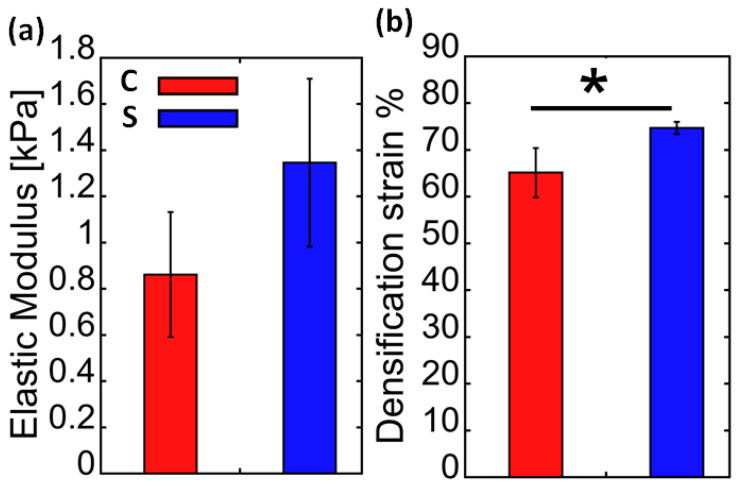
Comparison of elastic modulus (**a**) (compression) (MPa) and (**b**) densification strain (%) of bromotyrosine-containing (S, blue) and bromotyrosine-free (C, red) chitinous scaffolds isolated from *A. aerophoba* demosponge. * *p* < 0.05.

**Figure 4:**
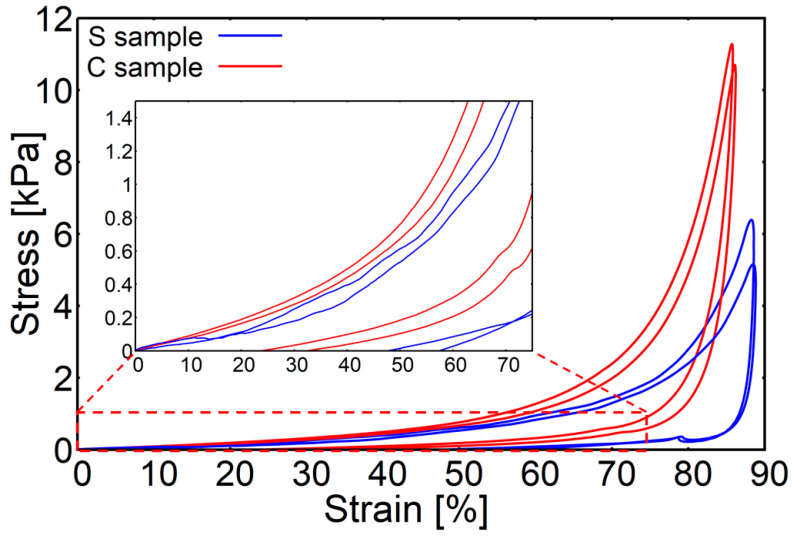
Loading–unloading hysteresis curves of the investigated chitinous scaffolds isolated from *A. aerophoba* demosponge. S samples (bromotyrosine-containing, blue), C samples (bromotyrosine-free, red).

The elastic moduli characterising the samples were determined (Figure 3a). The elastic modulus of the S (bromotyrosine-containing) samples is slightly higher than that of the C (bromotyrosine-free) samples; however, the difference remains to be statistically non-significant. The collapse stages of C and S samples look distinctively different. The modulus–strain curve of the C sample is much smoother than in the case of the S samples. One can see that the stress notably increased when both samples of the scaffolds were compressed to more than 60% strain, which may indicate the densification stage presence. The smoothness of the tangent modulus–strain curves more explicitly manifests the densification of the scaffolds under study (Figure 2). One can see from Figure 3b that in the case of the C samples, the densification strain is significantly lower than in the case of the S samples. In Figure 4, stress–strain hysteresis curves are presented. Both of the investigated specimen groups were permanently distorted; however, they still had some residual ability to reshape. It is worth emphasizing that in the case of the S samples, the deformation is irreversible to a greater extent than in the case of the C samples (Figure 4), which could suggest that a significant number of sponge-like struts of S samples lost their continuity.

### 2.2. Nanoindentation

**Figure 5:**
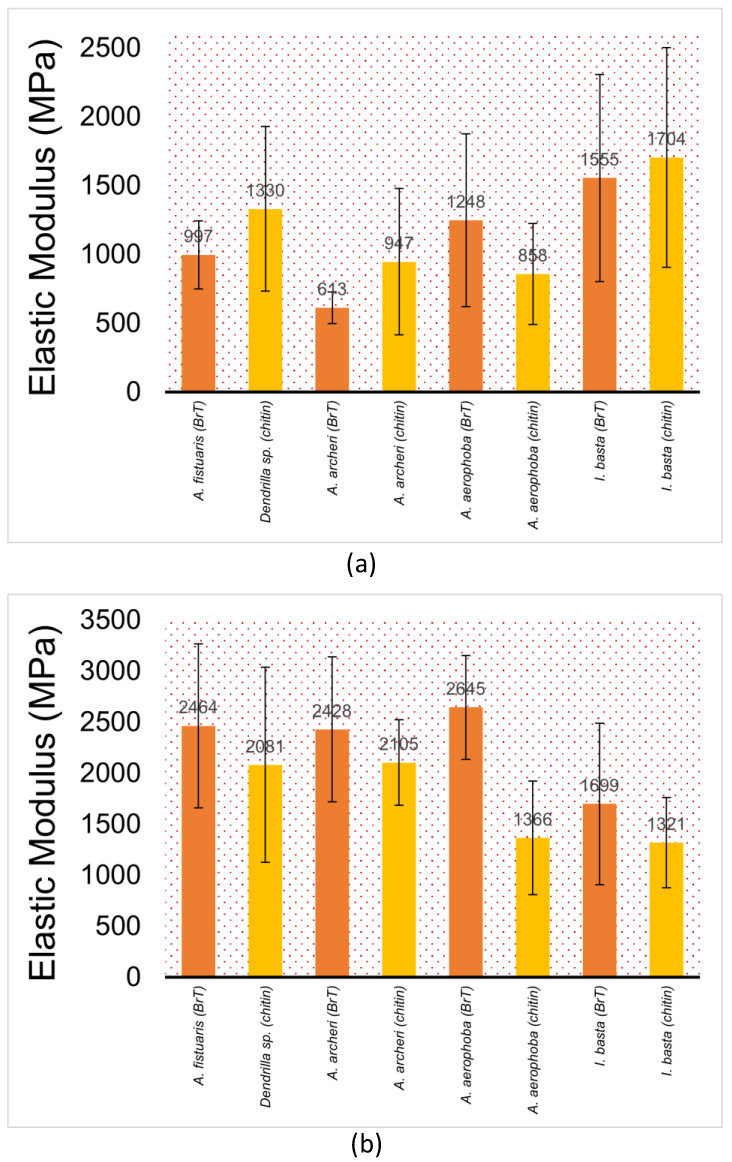
Elastic modulus (nanoindentation) in megapascals (MPa) of (**a**) 10 µm sections; (**b**) 1 µm sections of selected demosponges under study (from left to right: *A. fistularis*, *Dendrilla* sp., *A. archeri*, *A. aerophoba*, *Ianthella basta*) ± standard deviation. “BrT” denotes bromotyrosine-containing (orange). “Chitin” denotes sponge samples where bromotyrosines have been chemically removed (yellow).

Figure 5 shows the results of the elastic modulus (nanoindentation) for the decellularised chitinous fibres isolated from the selected demosponge.

**Figure 6:**
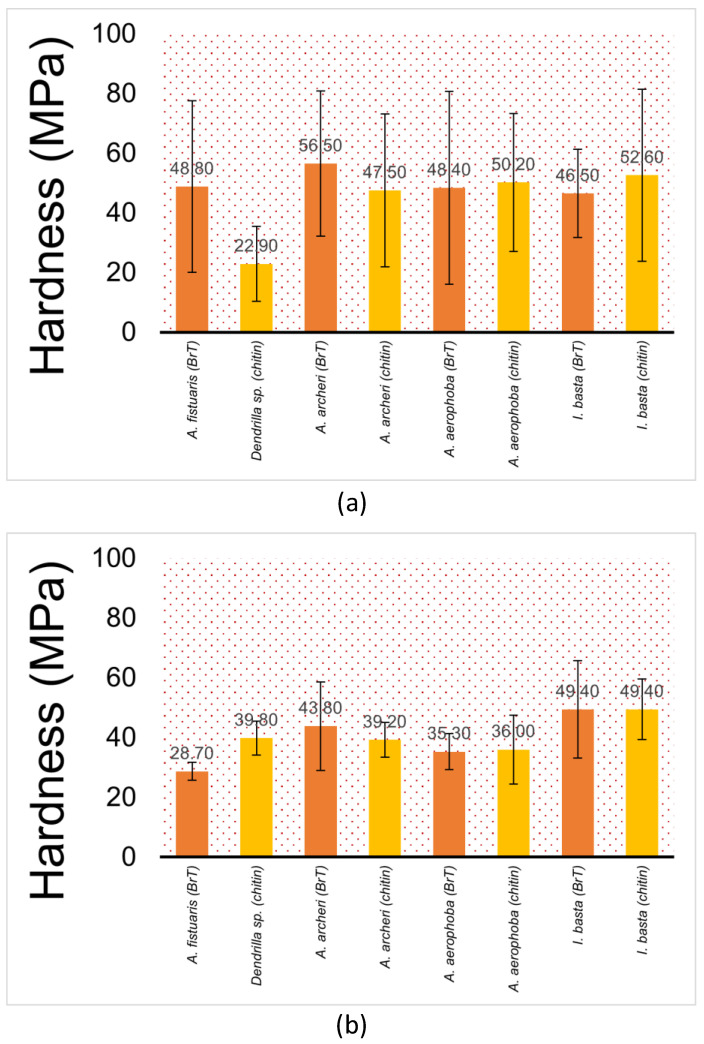
Hardness (nanoindentation) in megapascals (MPa) of (**a**) 10 µm sections; (**b**) 1 µm sections of selected decellularised fibres (from left to right: *A. fistularis, Dendrilla* sp., *A. archeri*, *A. aerophoba*, *I. basta*) ± standard deviation. “BrT” denotes bromotyrosine-containing (orange). “Chitin” denotes sponge samples where bromotyrosines have been chemically removed (yellow).

**Figure 7:**
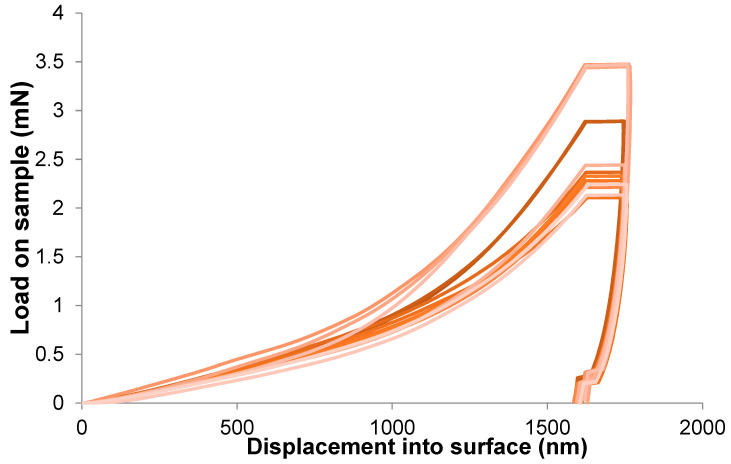
Indentation hysteresis curves for measurements on chitinous fibres of *A. aerophoba* demosponge origin.

The value of the elastic modulus changes between samples by about four times, and the hardness value of the hardest sample (i.e., *A. archeri*.) is about 60% higher than for the sample with the lowest hardness (i.e., *Dendrilla* sp.) (Figure 6). This can be influenced by the dispersion of fibre sizes and the natural way of formation. Moreover, examining the fibres from a single sample, we observed significant scatter in the results between them. The standard deviations of the arithmetic mean have values ranging from 20 to 50% of the measured value. This is due to the high inhomogeneity of the tested material. Figure 7 presents a summary of the nanoindentation hysteresis curves for one type of sample (several *A. aerophoba* fibres).

### 2.3. Scanning Electron Microscopy 

**Figure 8:**
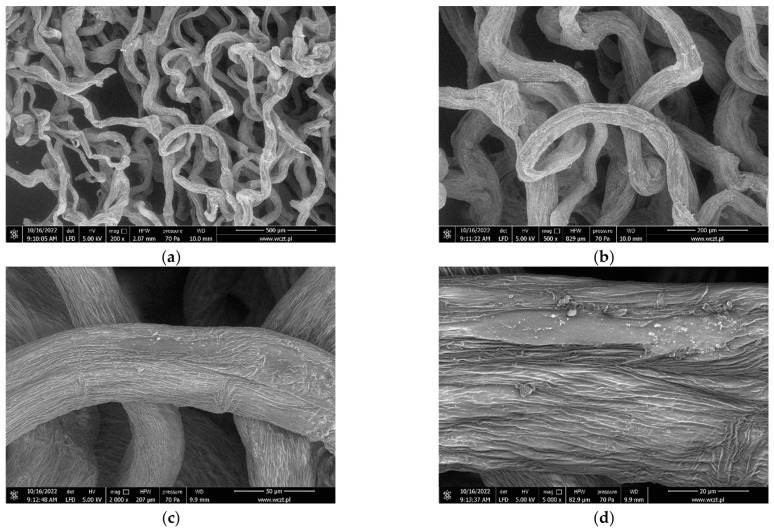
SEM images of the cell-free *A. aerophoba* chitin fibre scaffold’s fibre after decellularisation (**a**) ×200, 500 µm; (**b**) ×500, 200 µm; (**c**) ×2000, 50 µm; and (**d**) ×5000, 20 µm.

Figure 8 shows SEM images of mechanically pressed chitinous *A. aerophoba* sponge fibre samples at different magnifications: ×200, ×500, ×2000, and ×5000. The fibres exhibit random orientation and have a tendency to curve. Signs of shrinkage due to drying are visible. The average fibre diameter is around 59 ± 25.1 µm and varies greatly across a single fibre due to dehydration and uneven shrinkage. The porosity of the analysed samples was determined to be around 60% (pore size: 225 ± 96 µm).

### 2.4. Digital Optical Microscopy 

**Figure 9:**
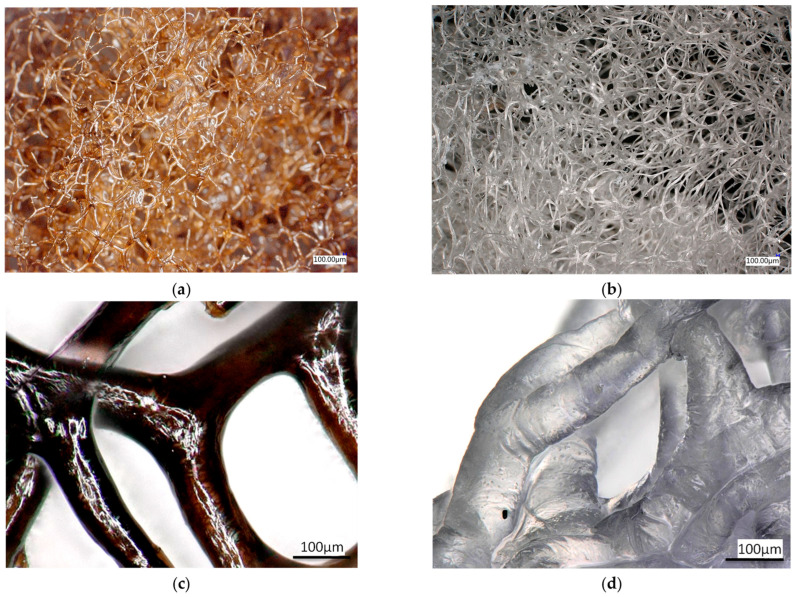
Digital microscopy images of the *A. aerophoba* chitin fibre scaffold after decellularisation. (**a**,**c**) Bromotyrosine-containing chitin scaffold, featuring a 3D porous network of microfibers. (**b**,**d**) Bromotyrosine-free chitin scaffold, featuring the same 3D porous network of microfibers.

Figure 9 shows bromotyrosine-containing (pigmented) and bromotyrosine-free skeletal samples isolated from an *A. aerophoba* demosponge. The bromotyrosine-containing sample in Figure 9a,c shows a 3D porous microfibre architecture with characteristic brownish pigmentation [25]. Diverse bromotyrosines localised within the skeletal fibres of the sponge *A. aerophoba* have been already identified by us previously [2,10]. We also reported the elemental content of such bromotyrosine-containing and cell-free chitinous skeletal fibres of this demosponge species analysed using EDX [42]. Not only Br, but also S, Cl, and traces of Ca have been identified by Nowacki and co-workers [42].

Bromotyrosine-free samples in Figure 9b,d show a similar architecture; however, as a result of alkali treatment [25], the microfibres have lost pigmentation and appear translucent. This is typical for isolated chitinous fibre-based scaffolds reported previously for other verongiid species [13].

## 3. Discussion

Identifying subsequent stages of the compression of cellular materials that follow nonlinear elastic stress–strain behaviour could be facilitated when analysing the tangent modulus–strain curve instead of the stress–strain curve [43]. The initial trend of the compression behaviour is related to the adjustment and stabilization phases of the sample-vice system. This section of both considered curves is usually quite disturbed and does not indicate the mechanical behaviour of the spongious structure. Afterwards, usually three distinct regions can be identified [44]:

1. At a low-stress level, an elastic stage can be observed. The spongious structure is deformed, but it is still structurally stable. The stresses transferred between the spongious struts are insufficient to induce permanent structural modifications. In this region, a modulus peak is usually observed, indicating the transition from the stable phase toward the subsequent collapse stage;
2. During the collapse stage, the characteristic plateau in the case of both of the considered curves is present. The plateau is associated with the collapse of the pores. A series of local collapses percolate through the structure at some critical stress. In particular, for an elastic foam, the plateau is due to elastic buckling, whereas, in the case of elastoplastic foams, it is due to the formation of plastic hinges [44]. Depending on the compressive mechanical behaviour of the cellular material, the plateau region can be characterised by a flat or slightly increasing slope stress plateau;
3. When the pores’ closure is almost completed, spongious struts begin to interact together whereby a rapid increase of stress takes place. At the same time, an abrupt increase in the modulus is observed. This last region of the curve is called the densification stage. When analysing the tangent modulus–strain curve, one can observe that its densification region is smoother than the collapse region, which facilitates the accurate discrimination between them [41].

The mechanical properties of chitinous scaffolds (Figure 9) derived from Verongiida sponges have not been experimentally studied in the past. However, it was reported by Machałowski and co-authors [11] that the verongiid demosponge *A. fistularis* may show a compressive theoretical modulus of 0.5 kPa, which is comparable with the results presented in this study (Figure 3).

The mechanical properties of poriferan skeletons [45] and sponge-like materials of non-poriferan origin [46] are rarely studied comparatively, and only a handful of studies exist [47,48,49]. These can be contrasted by the type of organic matrix exhibited, such as proteinaceous spongin-based skeletons of commercial bath sponges [48], collagen sponges [50], wood sponges [51], as well as cellulose-based Luffa fruit (i.e., *Luffa aegyptiaca*) sponges [49]. A direct comparison between the mechanical properties of the sponges (Porifera) investigated in this study is not possible due to significant differences; however, as an example, *Rhopaloeides odorabile* marine sponges with spongin skeletons studied by Louden and co-workers [48] showed an elastic modulus of less than one megapascal (838.7 ± 53.5 kPa). On the contrary, cellulose-containing sponges isolated from the Luffa fruit, for example, showed a Young’s modulus in the range 2–12 MPa, which is still well below the results presented in our study. This further confirms the unique nature and superior mechanical properties of the chitinous Verongiida demosponges under study. For example, the elastic modulus (nanoindentation) of the *A. aerophoba* demosponge skeleton, still containing bromotyrosines, is approximately 2.6 GPa (Figure 5), which is half of the bush crickets’ *Mecopoda elongata* acoustic tracheae (5.2 GPa) [29] and scorpion (*Scorpio palmatus*) tarsus exoskeleton (5.4 GPa) [49]. However, this value falls within the range of, for example, chitin films [28,52,53] but below, for example, crustacean nanofibres isolated from the American lobster (*Homarus americanus)* (Young’s modulus: 7.3 GPa) [54]. Such variations in the elastic modulus may be species-specific and are linked to different hierarchical arrangements and the chemical interplay between chitin and corresponding proteins as well as pigments [19].

A clear trend can be observed from Figure 3, Figure 5 and Figure 6 where the elastic modulus is influenced by whether the 3D scaffold sample contains bromotyrosines or is in the form of isolated, pigment-free chitin. A reduction in the elastic modulus is observed with chitin samples that have undergone alkali treatment [25] with respect to the extraction of all bromotyrosines [9]. This phenomenon is almost universally observed across all the chitin-based samples assessed in this study. However, this trend is not statistically significant.

Furthermore, another interesting phenomenon is observed: the hardness of sponge chitin samples that have undergone alkali treatment is relatively unchanged, which is a clear form of evidence that this kind of fibrous chitin is solely responsible for the material hardness observed in the samples studied. On the other hand, bromotyrosines in the skeletal chitinous fibres of the investigated sponges enhance their elasticity; however, they do not significantly contribute to their hardness. This can be explained by either the O-linked glycosylation bonding of bromotyrosines or hydrogen-bonding between chitin and bromotyrosines, or a mixture of both. Nonetheless, such a hypothesis would require future studies and confirmation by Nuclear Magnetic Resonance (NMR) spectroscopy.

The chitin samples of verongiid origin studied here undergo shrinking due to dehydration; however, they also undergo swelling once immersed in an aqueous medium [13]. The scaffolds studied here also exhibit “memory foam” properties, which is an attractive property from a tissue engineering point of view [55]. Memory foam properties can be explained by the rotationally flexible hydrogen conformation between the chitin chains [56].

The 3D chitinous scaffolds of verongiid origin exhibit typical hysteresis curves (Figure 4 and Figure 7) for brittle materials [57], with a sharp critical failure. However, upon the loss of bromotyrosines from the chitinous scaffold, a slight reduction in elasticity has been observed. The brittleness of the material is also confirmed by a very narrow elastic stage (Figure 2). Brittleness is very slightly more pronounced in the S samples (bromotyrosine-containing), which may be due to protein-reinforced matrix, i.e., chitin-bromotyrosine bonding. For example, previously [25], dibromotyrosines have been reported as crosslinking agents in cuticular structures of the Atlantic horseshoe crab (*Limulus polyphemus*) [58] as well as within scleroproteins found in the large whelk *Buccinum undatum* [59]. It has been recently found that proteins also play a role in the elastic properties of protein-chitin composites in the squid species of *Loligo vulgaris* and upon whole protein removal results in reduced elasticity [60]. This is also similar in the case of bush cricket *Mecopoda elongate* where dityrosine compounds cross-link protein resilin that results in significantly improved elasticity of the single taenidia fibres comprising the acoustic trachea, which the authors attributed to “*structural optimization between compliance and rigidity*” [29].

Nonetheless, renewable nanofibrillated cellulose (NFC) and nanofibrillated crustacean chitin (NFCh) nanoparticles comparable to chitinous sponge scaffolds were recently produced by wet-stretching and studied for mechanical properties as a function of macrofibre alignment where the investigating authors found that cellulose (elastic modulus: 33.7 GPa) outperformed chitin (elastic modulus: 12.6 GPa) and argued that this can be attributed to the higher axial modulus of cellulose I over α-chitin [61]. This study shows that the degree of alignment of microfibres can strongly influence the mechanical properties of chitin (see [62] for overview), which may also influence the mechanical properties of poriferan chitin observed in the present study.

It could be further postulated that based on the percentage reduction in the elastic modulus (Figure 5), it can be assumed with reasonable confidence that bromine and, therefore, protein content may be the highest in *A. aerophoba* (modulus reduction upon de-pigmentation, 48.35%), followed by *I. basta* (22.24%) and *A. archeri* (13.30%), which showed the least reduction upon de-pigmentation.

The mechanical properties observed in this study under wet conditions may not be suitable for immediate physiological load bearing; however, their dehydrated counterparts are comparable to human skin [32] and spongy bone [33]. This can explain why chitinous scaffolds of verongiid origin have already shown excellent and promising results in tissue engineering with respect to chondrocytes, cardiocytes, adipocytes, and diverse human mesenchymal stromal cells [6,7,11,63,64,65]. Nonetheless, bromotyrosine-containing skeletal matrices, such as those from *A. aerophoba* demosponge (Figure 9a,c), also have a great potential to be used as chemical catalysts and as templates which interact with metal ions through interaction with bromine and could serve in applications such as potential AgBr water filtration systems that have been recently investigated by Machałowski et al. [66]. Therefore, understanding the mechanical properties of these multiphase biomineralized [67] 3D constructs is quite essential.

We suggest that comparative studies on the mechanical properties of chitin fibres isolated from invertebrates, such as corals [68,69], coralline algae [70,71], as well as spiders [72,73] should be carried out in the near future, too.

## 4. Materials and Methods

### 4.1. Sample Preparation

Selected sponge samples (*Dendrilla* sp.—order Dendroceratida and *Aplysina fistuaris*, *Aplysina archeri*, *Aplysina aerophoba*, *Ianthella basta*—order Verongiida) purchased from INTIB GmbH and BromMarin GmbH, Freiberg, Germany were processed into two groups: bromotyrosine-containing (S) (decellularised) and chitin (C) (decellularised and bromotyrosine-free). Isolation of chitin scaffolds is described in more detail in [74]. Subsequently, the samples were stored in deionised water for compression testing and dried and embedded in a resin/sectioned (1 µm and 10 µm) for nanoindentation testing.

### 4.2. Monotonic Compression Test

The mechanical behaviour of specimens was investigated through monotonic compression tests. Hexahedral specimens with the approximate size of 10 mm × 10 mm × 4 mm were prepared. Monotonic compression tests were performed by means of a Q800 (TA Instruments, New Castle, DE, USA) instrument equipped with compression clamps enabled for testing in submersion. Specimens preloaded to 0.001 N were tested in native solution (deionised water, 10% methanol) and compressed at the constant strain rate of 5%/min. Following this, the unloading cycle was realized with the same strain rate. Tangent modulus–strain curves were determined based on the slope of the stress–strain curve at any specified strain. Experimental data were statistically analysed through one-way ANOVA and Tukey’s multiple pairwise comparisons test calculated by Origin 8. A *p*-value of 0.05 was considered to be significantly different. Data are expressed as mean ± standard deviation (*n* = 4).

### 4.3. Nanoindentation

Nanomechanical measurements were performed with an Agilent G200 nanoindenter. A DCMII measurement head was used, performing indentations with a maximum depth of 1600 nm. CSM mode was used during the measurements. The indenter used was made of diamond and had a Berkovich-type geometry. The instrument was calibrated before the measurement using the Oliver–Pharr method [75]. Measurements were made on fibres immobilized in resin, at the centre of the fibre cross-section. Each fibre type was analysed by indentation at several locations for several fibres (*n* = 10) in a given series. The reported errors of the determined parameters are the standard deviations of the obtained results. Experimental data were statistically analysed through one-way ANOVA and Tukey’s multiple pairwise comparisons test. A *p*-value of 0.05 was considered to be significantly different. Data are expressed as mean ± standard deviation (*n* = 10).

### 4.4. Scanning Electron Microscopy

The analyses were performed using the scanning electron microscope Quanta 250 FEG (FEI Ltd., Prague, Czech Republic). The samples were dried and pressed prior to the analyses.

### 4.5. Digital Optical Microscopy

The samples were observed with the advanced imaging systems consisting of a VHX-6000 digital microscope (Keyence, Osaka, Japan) and VH-Z20R zoom lenses (magnification up to 200×) as well as a Keyence VHX-7000 digital optical microscope with zoom lenses VHX E20 (magnification up to 100×) and VHX E100 (magnification up to 500×) (Keyence, Osaka, Japan).

### 4.6. Measurment of Porosity, Pore Size, and Fibre Diameter

The program ImageJ was used in this study in order to conduct the different image analysis techniques for calculating the porosity, pore size, and fibre diameter. The analyses were performed on electron micrographs obtained from the SEM experiments. Briefly, the software was calibrated prior to the measurement by using the scale on the electron micrographs (Figure 8). Subsequently, the binary threshold was adjusted, and the readings were taken for the porosity measurements. Similarly, the fibre diameter and pore size were also determined using the calibrated scale and respective functionality in the software.

## 5. Conclusions

We conclude that the combined analyses of monotonic compression and nanoindentation tests show a clear, but statistically insignificant trend that the removal of bromotyrosines from chitin scaffolds results in a reduced elastic modulus, however, a relatively unaffected hardness of the 3D scaffold. This is irrespective of the sample testing condition. Our study provides further evidence that the hardness observed with the scaffolds is imparted mainly by the specificity of sponge chitin. The presented mechanical properties of the natural 3D chitin scaffolds isolated from the cultivated *A. aerophoba* and other sponges are crucial for their further use in tissue engineering.

## Figures and Tables

**Table 1:** Comparison of mechanical properties between different biomaterials.

	Biomaterial	Elastic Modulus (MPa)	Ultimate Tensile Strength (MPa)	Source
Chitin	Bush crickets’ acoustic tracheae	5200	-	[29]
	Sheep crab exoskeleton (wet)	518 ± 72	31.5 ± 5.4	[30]
	Sheep crab exoskeleton (dry)	764 ± 83	12.9 ± 1.7	
	Commercial flake chitin films (solvent-casting)	1240–3650	38–60	[28]
	Philippine blue swimming crab chitin (solvent-casting)	-	44.22	[31]
Human Tissues	Human skin (back)	98.97 ± 97	27.2 ± 9.3	[32]
	Human femoral cancellous bone	441	6.8	[33]

## Data Availability

The original data presented in the study are included in the article; further inquiries can be directed to the corresponding author.
